# Middle Pleistocene protein sequences from the rhinoceros genus *Stephanorhinus* and the phylogeny of extant and extinct Middle/Late Pleistocene Rhinocerotidae

**DOI:** 10.7717/peerj.3033

**Published:** 2017-03-14

**Authors:** Frido Welker, Geoff M. Smith, Jarod M. Hutson, Lutz Kindler, Alejandro Garcia-Moreno, Aritza Villaluenga, Elaine Turner, Sabine Gaudzinski-Windheuser

**Affiliations:** 1 Department of Human Evolution, Max Planck Institute for Evolutionary Anthropology, Leipzig, Germany; 2 BioArCh, Department of Archaeology, University of York, York, UK; 3 MONREPOS Archaeological Research Centre and Museum for Human Behavioural Evolution, RGZM, Neuwied, Germany; 4 Department of Anthropology, University of California Davis, Davis, CA, USA; 5 Department of Pre- and Protohistoric Archaeology, Institute of Ancient Studies, Johannes-Gutenberg Universität Mainz, Mainz, Germany; 6 Current affiliation: Department of Paleobiology, Smithsonian Institution, Washington, D.C., USA

**Keywords:** Palaeoproteomics, Phylogenetics, Rhinocerotidae, Ancient proteins, Stephanorhinus

## Abstract

**Background:**

Ancient protein sequences are increasingly used to elucidate the phylogenetic relationships between extinct and extant mammalian taxa. Here, we apply these recent developments to Middle Pleistocene bone specimens of the rhinoceros genus *Stephanorhinus*. No biomolecular sequence data is currently available for this genus, leaving phylogenetic hypotheses on its evolutionary relationships to extant and extinct rhinoceroses untested. Furthermore, recent phylogenies based on Rhinocerotidae (partial or complete) mitochondrial DNA sequences differ in the placement of the Sumatran rhinoceros (*Dicerorhinus sumatrensis*). Therefore, studies utilising ancient protein sequences from Middle Pleistocene contexts have the potential to provide further insights into the phylogenetic relationships between extant and extinct species, including *Stephanorhinus* and *Dicerorhinus*.

**Methods:**

ZooMS screening (zooarchaeology by mass spectrometry) was performed on several Late and Middle Pleistocene specimens from the genus *Stephanorhinus*, subsequently followed by liquid chromatography-tandem mass spectrometry (LC-MS/MS) to obtain ancient protein sequences from a Middle Pleistocene *Stephanorhinus* specimen. We performed parallel analysis on a Late Pleistocene woolly rhinoceros specimen and extant species of rhinoceroses, resulting in the availability of protein sequence data for five extant species and two extinct genera. Phylogenetic analysis additionally included all extant Perissodactyla genera (*Equus*, *Tapirus*), and was conducted using Bayesian (MrBayes) and maximum-likelihood (RAxML) methods.

**Results:**

Various ancient proteins were identified in both the Middle and Late Pleistocene rhinoceros samples. Protein degradation and proteome complexity are consistent with an endogenous origin of the identified proteins. Phylogenetic analysis of informative proteins resolved the Perissodactyla phylogeny in agreement with previous studies in regards to the placement of the families Equidae, Tapiridae, and Rhinocerotidae. *Stephanorhinus* is shown to be most closely related to the genera *Coelodonta* and *Dicerorhinus*. The protein sequence data further places the Sumatran rhino in a clade together with the genus *Rhinoceros*, opposed to forming a clade with the black and white rhinoceros species.

**Discussion:**

The first biomolecular dataset available for *Stephanorhinus* places this genus together with the extinct genus *Coelodonta* and the extant genus *Dicerorhinus*. This is in agreement with morphological studies, although we are unable to resolve the order of divergence between these genera based on the protein sequences available. Our data supports the placement of the genus *Dicerorhinus* in a clade together with extant *Rhinoceros* species. Finally, the availability of protein sequence data for both extinct European rhinoceros genera allows future investigations into their geographic distribution and extinction chronologies.

## Introduction

Two rhinoceros genera were present in Western Europe during the Late and Middle Pleistocene, *Coelodonta* (Bronn, 1831) and *Stephanorhinus* (Kretzoi, 1942), formerly included in the genus *Dicerorhinus* (Fortelius, Mazza & Sala, 1993). For *Coelodonta*, ancient DNA studies of the enigmatic woolly rhinoceros all confidently place this genus together with the Sumatran rhinoceros (Orlando et al., 2003; Willerslev et al., 2009; Yuan et al., 2014), as previously suggested based on skeletal morphology and biogeography (Nowak, 1999). In addition, both the Sumatran rhinoceros and the woolly rhinoceros have a hairy coat. No molecular data for the genus *Stephanorhinus* is currently available although a sister-clade relationship with *Coelodonta* has often been suggested based on morphology (Deng et al., 2011; Groves, 1983). Relationships between (successive) species of *Stephanorhinus* have remained obscure. A general view proposes that *Stephanorhinus kirchbergensis* (or *Dihoplus kirchbergensis* following Deng et al., 2011) falls outside a clade comprising *Suncus etruscus*, *Stephanorhinus hundsheimensis*, and *Stephanorhinus hemitoechus* (Deng et al., 2011; Fortelius, Mazza & Sala, 1993). A recent cladistics analysis indicated *Stephanorhinus hemitoechus* as a sister group to the genus *Coelodonta* (Deng et al., 2011). The genus *Stephanorhinus* went extinct in the middle Late Pleistocene, although an exact chronology has not been established, while the woolly rhinoceros survived locally to 14 ka before present (Stuart & Lister, 2012).

Analysis of Rhinocerotidae morphology (Cerdeño, 1995; Fortelius, Mazza & Sala, 1993; Groves, 1983; Loose, 1975; Nowak, 1999; Prothero, Manning & Hanson, 1986; Prothero & Schoch, 1989; Simpson, 1945) and Rhinocerotidae DNA sequences (Morales & Melnick, 1994; Price & Bininda-Emonds, 2009; Steiner & Ryder, 2011; Tougard et al., 2001; Willerslev et al., 2009) has provided insights into Rhinocerotidae evolution and systematics. Nevertheless, some differences remain in the phylogenetic placement of the genus *Dicerorhinus* when concerning generated phylogenetic trees alone. Proposed phylogenies are commonly divided between (1) those placing *Dicerorhinus sumatrensis* (Sumatran rhinoceros) as a sister group to the genus *Rhinoceros* (*Rhinoceros sondaicus*, Javan rhinoceros; and *Rhinoceros unicornis*, Indian rhinoceros), (2) those placing *Dicerorhinus* with the black and white rhinoceroses (respectively, *Diceros bicornis*, black rhinoceros; *Ceratotherium simum*, southern white rhinoceros, and *Ceratotherium cottoni*, northern white rhinoceros; Groves, Fernando & Robovský, 2010; but see Harvey et al., 2016), or (3) those resulting in a polytomy at the base of extant Rhinocerotidae consisting of three separate lineages (the black and white rhinoceroses, the Sumatran rhinoceros, and the Javan and Indian rhinoceroses). Each of these has been supported by one or several genetic studies focusing on mitochondrial DNA (Morales & Melnick, 1994; Tougard et al., 2001; Willerslev et al., 2009; Yuan et al., 2014). Conflicting phylogenetic results seem to arise from differences in mitochondrial locus selection and rapid divergence of the three lineages during the Oligocene (Willerslev et al., 2009). The additional use of nuclear DNA in combination with selected mitochondrial gene sequences has also resulted in conflicting results on the phylogenetic position of *Dicerorhinus* in relation to other extant genera (Price & Bininda-Emonds, 2009; Steiner & Ryder, 2011). The differing positioning of *Dicerorhinus* in these two studies might simply be due to differences in the mitochondrial genes included in each analysis, as discussed above (Willerslev et al., 2009). Molecular sequences of additional, now extinct, species of rhinoceros have the potential to contribute to our understanding of the relationships among these mammals, as it has done in the past for other mammals by utilising ancient DNA or ancient protein sequences (Der Sarkissian et al., 2015; Kistler et al., 2015; Lister et al., 2005; Welker et al., 2015a).

Here, we apply palaeoproteomic analysis of ancient protein sequences to provide additional molecular data on the relationship between the genus *Stephanorhinus*, the genus *Coelodonta* (*Coelodonta antiquitatis*, the woolly rhinoceros) and five extant rhinoceros species. Previously, palaeoproteomic analysis of the collagen type I (COL1) heterotrimer has been successful in providing molecular data on the phylogenetic relationship between extinct and extant mammalian taxa (Buckley, 2013; Cleland et al., 2016; Rybczynski et al., 2013; Welker et al., 2015a). Such studies utilised temperate and tropical samples of Late Pleistocene age, the *Macrauchenia* specimens from Welker et al. (2015a) being a possible Middle Pleistocene exception. Other studies have reported the presence of complex or reduced palaeoproteomes in Middle Pleistocene bone specimens from temperate or permafrost conditions without using the retrieved sequence data for phylogenetic purposes (Orlando et al., 2013; Wadsworth & Buckley, 2014). Previous palaeoproteomic phylogenetic studies have focused their efforts on the COL1 protein because of its dominance within the bone proteome and its resilience against degradation (Buckley, 2015; Cleland et al., 2016; Rybczynski et al., 2013; Welker et al., 2015a). The COL1 heterotrimer, composed of two collagen alpha-1(I) (*COL1α1*) amino acid sequences and one collagen alpha-2(I) (*COL1α2*) amino acid sequence, generally provides genus-level sequence information, with only limited cases where within-genus amino acid sequence variation is present in either of the two genes (for example, between Arctic fox and Red fox; *Vulpes lagopus* and *Vulpes vulpes*, respectively; Welker et al., 2016). Elsewhere, it has been noted that other collagens and non-collagenous proteins (NCPs) potentially provide more detailed phylogenetic information (Wadsworth & Buckley, 2014; Welker et al., 2016). Here, we utilise the phylogenetic information retrieved from Middle and Late Pleistocene COL1 sequences, including the incorporation of amino acid protein sequence data from other proteins besides COL1.

## Materials and Methods

### Protein extraction and analysis

Fourteen Pleistocene bone and dental specimens were screened for the presence of COL1 using successive ammonium-bicarbonate buffer extraction (Table 1; van Doorn, Hollund & Collins, 2011) and acid demineralisation on bone/dentine samples measuring roughly <30 mg (as in Welker et al., 2016). Both soluble and in-soluble COL1 components were analysed separately on a Bruker MALDI-TOF-MS to obtain COL1 fingerprints (Buckley et al., 2009). Four samples (*Stephanorhinus* sp.) derive from the Middle Pleistocene site of Schöningen 13II-4. These samples are associated with unique wooden spears (Thieme, 1997), and must be younger than the age of Schöningen 13I-1 (321 ± 6 ka; Richter & Krbetschek, 2015). The age of Schöningen 13II-4 might be very close to the age of 13I-1, however, and has been suggested to fall within the 337–300 ka/MIS9 time range. Botanical remains from this late interglacial lakeshore site suggest a steppic, open forest environment related with the spears (Urban & Bigga, 2015). Nine samples (either representing *Stephanorhinus* sp. or *Coelodonta antiquitatis*) derive from the Eemian site Neumark-Nord 2, the age of which is approximately 126 ± 6 ka (Sier et al., 2011). Neumark-Nord 2 represents a basin infill with hominin presence in relation to a semi-open environment (Britton et al., 2012; Gaudzinski-Windheuser et al., 2014; Pop & Bakels, 2015). Initial analysis of the faunal assemblage from the site suggests that hominins represent the sole accumulators, with limited in situ reorientation of bone specimens (García-Moreno et al., 2016; Kindler, Smith & Wagner, 2014). Rhinocerotidae found in relation to the archaeological find horizons at the site might represent either of three species known to be present within the wider Neumark-Nord landscape at the time, *Coelodonta antiquitatis*, *Stephanorhinus kirchbergensis*, and *Stephanorhinus hemitoechus* (van der Made, 2010). Bone specimens studied here could not be assigned to a specific rhinoceros taxon, and where hence included as representing Rhinocerotidae. Finally, a single woolly rhinoceros sample was analysed from Late Pleistocene deposits in Siberia, although an exact location or age is unknown (sample PMF40; Welker et al., 2016).

**Table 1:** Samples used in this study.

Sample name	Site	Inventory number	Tissue type	Morphological identification
PMF69	Modern^1^	RMNH.MAM.5738	Nasal cartilage	*Diceros bicornis*
PMF70	Modern^1^	ZMA.MAM.23879	Nasal cartilage	*Rhinoceros unicornis*
PMF71	Modern^1^	ZMA.MAM.7681	Nasal cartilage	*Rhinoceros sondaicus*
PMF72	Modern^1^	ZMA.MAM.539	Nasal cartilage	*Dicerorhinus sumatrensis*
PMF40	Siberia		Bone	*Coelodonta antiquitatis*
NN1	Neumark-Nord 2	2071-B3-NN2/2/9131	Bone	Rhinocerotidae
NN2	Neumark-Nord 2	2072-B3-NN2/2/9298	Bone	Rhinocerotidae
NN3	Neumark-Nord 2	2071-B3-NN2/2/9069	Bone	Rhinocerotidae
NN4	Neumark-Nord 2	2071-B2-NN2/2/8951	Dentine	Rhinocerotidae
NN5	Neumark-Nord 2	2071-B2-NN2/2/8951	Bone	Rhinocerotidae
NN6	Neumark-Nord 2	2071-B2-NN2/2/9002	Bone	Rhinocerotidae
NN7	Neumark-Nord 2	2120-B3-NN2/2/18229	Dentine	Rhinocerotidae
NN8	Neumark-Nord 2	2145-B1/2-NN2/2/15389	Dentine	Rhinocerotidae
NN9	Neumark-Nord 2	2086-B3-NN2/2/10316	Dentine	Rhinocerotidae
SCH1	Schöningen 13II-4	20114: 679/0-8	Bone	*Stephanorhinus* sp.
SCH2	Schöningen 13II-4	19066: 674/8-1	Bone	*Stephanorhinus* sp.
SCH3	Schöningen 13II-4	14527: 677/3-1	Bone	*Stephanorhinus* sp.
SCH4	Schöningen 13II-4	14522: 676/2-1	Bone	*Stephanorhinus* sp.

**Note:**

1 Collection Naturalis Biodiversity Centre, Leiden, the Netherlands.

After determination of the presence of endogenous COL1, two *Stephanorhinus* extracts were analysed using liquid chromatography-tandem mass spectrometry (LC-MS/MS) analysis. In short, peptides were trapped on a Pepmap μ-pre-column (Thermo Scientific) and separated on an EASY Spray PepMap UHPLC column with a 60 min gradient (Thermo Scientific) with direct injection into a Q-Exactive hybrid quadrupole-Orbitrap (Thermo Scientific) at the University of Oxford (as in Welker et al., 2015a, 2016).

Modern samples from four extant species were obtained from Naturalis Biodiversity Centre, Leiden, the Netherlands (Table 1). These samples derive from nasal cartilage, which formed a suitable target tissue while minimising damage to the bone morphology of the sampled skulls. COL1 from these four extant rhinoceros species was extracted and analysed in an identical way as explained above. COL1 fingerprints were previously obtained for the included extant specimens as well as the *Coelodonta* data (Welker et al., 2016). A published and annotated genome was available for the fifth extant rhinoceros species, the southern white rhinoceros (*Ceratotherium simum*; GenBank RefSeq accession GCF_000283155.1, number of protein sequences = 33,626).

LC-MS/MS .raw files were converted to .mgf using ProteoWizard (Chambers et al., 2012) and searched against the southern white rhinoceros protein reference set (downloaded 17/08/2015), including common contaminants, on PEAKS v.7 (Ma et al., 2003). Each search incorporated a set of >100 possible contaminating proteins including human keratins, bovine albumin, and porcine trypsin. Searches allowed for up to six modifications per peptide, with oxidation (M), hydroxylation (P), and deamidation (N/Q) suggested as variable modifications. Proteins were accepted when two unique peptides were identified and a protein score of −10lgP ≥ 20 was obtained. Peptide–spectrum matches were accepted with a false discovery rate equal to 1.0%, including for peptide–spectrum matches containing amino acid substitutions compared to the reference sequence. De novo matches were only included when the ALC was ≥50%, and inspected manually. Data for *Coelodonta* was previously obtained and re-analysed here following the above parameters (Welker et al., 2016).

An extraction blank was run alongside bone protein extracts, which remained empty of COL1, while other proteins present in this blank were removed from further analysis when also present in rhinoceros extracts (trypsin, keratins, and histones). In addition, blank runs were incorporated between LC-MS/MS runs containing Rhinocerotidae extracts to minimise the risk of sample carry-over (Demarchi et al., 2016). Furthermore, we excluded several proteins from further analysis that have been identified previously as contaminants in other publications, while matches to other non-rhinoceros proteins were facilitated by the inclusion of possible contaminating proteins (see above). Finally, we looked at deamidation frequencies for individual proteins identified in Pleistocene samples to support our interpretation of endogenous and contaminating proteins. To achieve this, we counted, per protein, the number of spectral matches containing deamidated glutamine and asparagine positions after bioinformatic analysis and divided this by the total number of spectra matching glutamine and asparagine positions (regardless of deamidated or non-deamidated status). Only proteins with a minimum number of three glutamine and/or asparagine positions covered by peptide–spectrum matches were included in cluster analysis (Mclust) to assign group membership (following Welker et al., 2016).

### Phylogenetic analysis

Collagen alpha-1(I) and alpha-2(I) sequences were concatenated, leucines converted into isoleucines, as these two amino acids are isobaric, and telopeptides removed from both. The COL1 (*COL1α1* and *COL1α2*) sequence obtained for SCH2 was removed from the alignment after it was demonstrated that this sequence was mainly composed of conserved regions (Fig. S1). For proteins other than COL1, peptides were included in phylogenetic analysis when at least one peptide from either of the specimens studied here contained an amino acid substitution differing from the southern white rhinoceros reference sequence (collagen alpha-1(III) [*COL3α1*], pigment epithelium-derived factor [*PEDF*], Alpha-2-HS-glycoprotein [*AHSG*]). Total alignment length comprised 2,089 amino acid positions, and included five extant rhinoceroses, the woolly rhinoceros, *Stephanorhinus* sp. (SCH3, possibly *kirchbergensis*), *Tapirus terrestris*, *Equus asinus*, *Equus caballus*, and *Equus hydruntinus. Bos primigenius* was used as an outgroup. Sequence data for the latter species was obtained from GenBank and previous publications (Welker et al., 2015a, 2016).

CIPRES Science Gateway (Miller, Pfeiffer & Schwartz, 2010) was used to conduct Bayesian phylogenetic analysis using MrBayes and maximum-likelihood phylogenetic analysis using RAxML. For both analyses, the alignment was partitioned by gene to allow variable substitution rates within the Dayhoff substitution model (selected after running PartitionFinderProtein; Lanfear et al., 2012). MrBayes was run for five million generations, with sampling every 500 generations, and the first 10% was discarded as burn-in. Inspection of the likelihood (LnL) effective sample size (ESS) in Tracer v.1.6.0 provided a value of >200 for all trees, and all other parameters, indicating that discarding 10% as burn-in was a sufficient number of removed chain steps. RAxML was run for 1,000 bootstrap iterations.

### Data availability

Pleistocene proteomic data generated for sample SCH3 and the woolly rhinoceros sample are available under ProteomeXchange accession number PXD005534. Proteomic data generated for the extraction blank are available under ProteomeXchange accession number PXD003208. Sequence data from the genera *Bos*, *Tapirus*, and *Equus* were taken from GenBank or directly from previous publications (Welker et al., 2015a, 2016). Table S1 contains the accession numbers or literature references for sequence availability per gene. Fasta File S1 contains the individual protein sequences for genes used in phylogenetic analysis (*COL1α1*, *COL1α2*, *COL3α1*, *PEDF*, and *AHSG*).

## Results

ZooMS (zooarchaeology by mass spectrometry) screening was successful for bone specimens from both Schöningen 13II-4 and Neumark-Nord 2. Two bone specimens from Schöningen 13II-4 were selected for LC-MS/MS analysis (samples SCH2 and SCH3), in addition to a woolly rhinoceros sample from Siberia (sample PMF40). The SCH2 COL1 sequence is very incomplete (30.3% of the COL1 sequences covered). Other than COL1, no typical proteins of the bone proteomes were observed in this sample. A MrBayes tree resulted in an erroneous position of this sample at the base of Rhinocerotidae (Fig. S1), instead of a position close to SCH3. Based on these observations, we decided to exclude this sample from further analysis.

**Figure 1:**
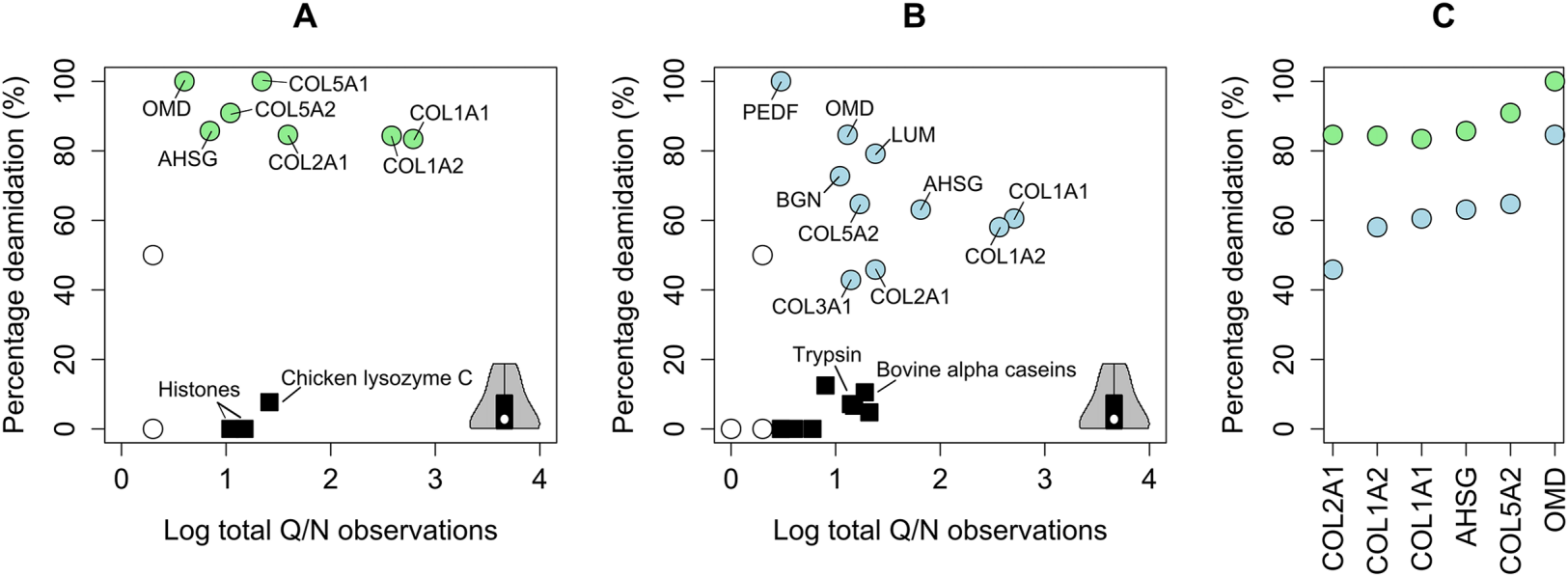
Deamidation frequency for identified Pleistocene proteins. (A) SCH3, *Stephanorhinus* sp. (B) *Coelodonta antiquitatis*. Open circles represent proteins with two or less glutamine/asparagine positions detected, and were not included in cluster analysis. Black squares represent contaminating proteins, and filled circled represent proteins endogenous to the SCH3 sample (green, A) or the woolly rhinoceros sample (blue, B). Grey inset indicates glutamine/asparagine deamidation for contaminating proteins in the modern Rhinocerotidae samples, depicted as violin plot. (C) Spectral deamidation frequency for five endogenous proteins with at least two glutamine/asparagine containing peptides present in both the SCH3 and the woolly rhinoceros sample. Circle colour corresponds to those in (A) *Stephanorhinus* sp. and (B) *Coelodonta antiquitatis*.

For the five included extant rhinoceroses, SCH3 and the woolly rhinoceros specimen, we obtained COL1 sequence coverage >90%. In addition to both COL1 alpha chains, we confidently identified five additional proteins for SCH3 (collagen alpha-1(II), collagen alpha-1(V) [*COL5α1*], collagen alpha-2(V) [*COL5α2*], *AHSG*, osteomodulin [*OMD*], and possibly nucleolin [*NCL*]). For the woolly rhinoceros sample, which is undated but presumably Late Pleistocene in age, we confidently identify ten proteins in addition to COL1 (collagen alpha-1(II) [*COL2A1*], *COL3α1*, collagen alpha-1(V), collagen alpha-2(V), *AHSG*, biglycan [*BGN*], *OMD*, lumican [*LUM*], *PEDF*, and chondroadherin [*CHAD*]). These proteins have elevated deamidation frequencies for glutamine and asparagine positions compared to known contaminants, proteins present in the extraction blank, and added proteins (trypsin) present in the same extracts (Figs. 1A and 1B). They are also absent from the extraction blanks, and represent proteins previously detected in (ancient) bone proteome studies (Cappellini et al., 2012; Wadsworth & Buckley, 2014).

Contaminants are represented by, among others, porcine trypsin, which is added during our proteomic workflow as the digestive enzyme, bovine alpha and beta caseins (alpha-S1-casein [*CSN1S1*], alpha-S2-casein [*CSN1S2*], beta-casein [*CSN2*], and chicken lysozyme C [*LYZ*]; Figs. 1A and 1B). These contaminants have deamidation frequencies below that observed for endogenous bone proteins. Furthermore, the deamidation frequency of these proteins is similar in Pleistocene and modern bone extracts (see grey violin in Figs. 1A and 1B).

Furthermore, deamidation frequencies for the five proteins present in both the SCH3 and the woolly rhinoceros sample display elevated deamidation frequencies in the SCH3 sample (Fig. 1C). This is consistent with increased protein degradation for the Middle Pleistocene sample compared to the Late Pleistocene woolly rhinoceros sample, as also demonstrated by the decrease in protein diversity recovered. We could not compare deamidation values with modern extracts as the modern rhinoceros samples represented non-mineralised nasal cartilage. Extracts for modern rhinoceros samples contained up to 65 proteins. These include the proteins identified for SCH3 and the woolly rhinoceros sample. This overall reduction of proteome complexity is consistent with previous reports, which also demonstrate the prolonged survival of *AHSG* and *OMD* (Orlando et al., 2013; Wadsworth & Buckley, 2014).

For some proteins, all spectral matches to glutamines and asparagines are deamidated (expressed as 100% deamidation). Hypothetically, these could indicate amino acid substitutions towards glutamic acid and aspartic acid amino acids, respectively. We consider them to represent deamidated glutamines and asparagines because of (1) the overall high frequency of deamidation in our dataset, (2) the increase of deamidation in the older sample compared to the younger sample, consistent with theoretical expectations of protein degradation, and (3) the absence of glutamine and asparagine substitutions to glutamic acid and aspartic acid, respectively, in our comparative protein sequence alignment for species whose protein sequences are obtained through genomic databases (*B. primigenius*, *Equus caballus*, and *Ceratotherium simum*). In other words, despite the evolutionary divergence between these three species, there are no instances of glutamine to glutamic acid and asparagine to aspartic acid substitutions among the endogenous proteins detected in our study. Nevertheless, future research should look into methodological developments to separate completely deamidated glutamine or asparagines positions from glutamine to glutamic acid and asparagine to aspartic acid substitutions.

**Figure 2:**
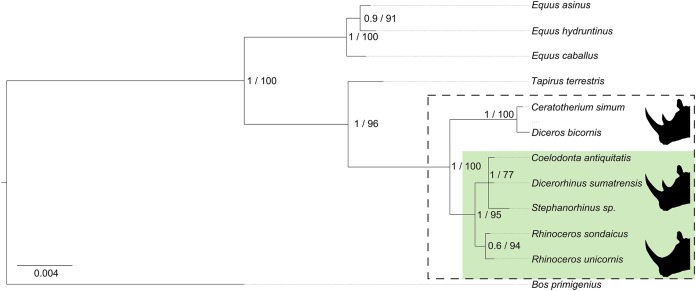
Bayesian phylogenetic tree of studied Perissodactyla. Rhinoceros species present in Eurasia are highlighted by the green box. Silhouettes indicate the number of horns. Node numbers indicate Bayesian probability (0–1)/maximum likelihood (0–100%). *B. primigenius* was used as an outgroup.

Our phylogenetic analysis resulted in a phylogeny with high node support (at least either RAxML or MrBayes ≥90%) for all nodes and an identical topology between Bayesian and RAxML methods (Fig. 2). The relationships among the three major Perissodactyla families (Equidae, Tapiridae, and Rhinocerotidae) are resolved in agreement with other studies indicating a basal split between Hippomorpha (Equidae) and Ceratomorpha (Tapiridae and Rhinocerotidae; Steiner & Ryder, 2011). We note that the relationship between the included Equidae has support for separate caballine and non-caballine horse clades, again in line with previous studies (Orlando et al., 2013; Steiner & Ryder, 2011). Within Rhinocerotidae, the separate clades of the white and black rhinoceroses and that of the two *Rhinoceros* species are both supported. Relationships between the extinct *Coelodonta* and *Stephanorhinus* genera and the extant *Dicerorhinus* genus are not resolved, although the three species together form a single clade. This is in agreement with (ancient) mtDNA evidence for *Coelodonta* + *Dicerorhinus* presented previously (Willerslev et al., 2009).

Our study is the first to report molecular evidence supporting an association between the genus *Stephanorhinus* and *Coelodonta* + *Dicerorhinus*, as suggested previously based on skeletal morphology (Groves, 1983; Nowak, 1999). The clade comprised of the genus *Dicerorhinus* and the two studied extinct genera groups with that of the other Asian rhinoceroses (*Rhinoceros* sp.). Following our phylogeny, the extant African rhinoceros species form the most basal clade within the (extant) Rhinocerotidae, which is in agreement with a recent morphological study (Deng et al., 2011).

## Discussion

We demonstrate the presence of phylogenetically informative ancient protein sequences in the Middle Pleistocene of Europe, without the need to resolve towards large bone samples sizes (<30 mg). This is in line with observations made previously for the Late Pleistocene (Welker et al., 2016). In general, ancient proteomes will decrease in complexity over time. The resilience of COL1 to degradation results in a dominance of this protein compared to other proteins (Wadsworth & Buckley, 2014), an observation which is replicated by the data obtained in this study. Other proteins do survive, however, including additional collagens and NCPs, even in bone samples from Middle Pleistocene open-air sites. These additional proteins have so far received little attention in phylogenetic studies based on ancient protein sequence data, partly because sequence coverage for such proteins is low.

Our study confirms the proposed close phylogenetic relationship between the genera *Dicerorhinus*, *Stephanorhinus*, and *Coelodonta*, but the order of divergence of the three genera is unresolved. Previous genetic studies on the phylogenetic placement of extant rhinoceroses have been inconclusive in the placement of the genus *Dicerorhinus*. Although aDNA studies demonstrated that the extinct woolly rhinoceros is most closely related to the extant Sumatran rhinoceros, they did not improve the relationships among extant rhinoceroses concerning *Dicerorhinus* (Willerslev et al., 2009). As highlighted in the introduction, the value of mtDNA studies for the Rhinocerotidae is limited, as different mitochondrial genes resolve the relationships among Rhinocerotidae in different ways (Willerslev et al., 2009). Therefore, it is not surprising that our phylogeny differs from some (Morales & Melnick, 1994) but not all (Orlando et al., 2003; Tougard et al., 2001) studies utilising mtDNA loci. The protein phylogenetic tree obtained in this study differs from one study utilising a combined mitochondrial and nuclear DNA dataset (Steiner & Ryder, 2011) but agrees with another in placing *Dicerorhinus* with *Rhinoceros* (Price & Bininda-Emonds, 2009). Studies focusing solely on nuclear biomolecular data might be favoured, however, given the problems associated with using Rhinocerotidae mtDNA loci.

**Figure 3:**
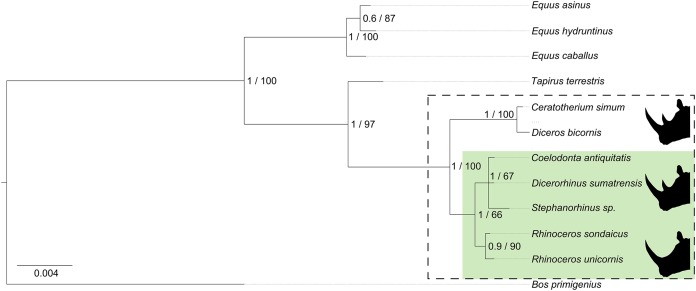
Extended Bayesian phylogenetic tree. Here, the alignment consists of proteomic sequence data generated in this study and the translated protein sequences from the genetic data generated in Steiner & Ryder (2011). Rhinoceros species present in Eurasia are highlighted by the green box. Silhouettes indicate the number of horns. Node numbers indicate Bayesian probability (0–1)/maximum likelihood (0–100%). *B. primigenius* was used as an outgroup.

To explore the consistency of our phylogenetic tree with the data generated by Steiner & Ryder (2011), we added the translated protein sequences from the nuclear sequence data generated in their study (total length = 3,917 amino acids, genes *BRCA1*, *EDNRB*, *KIT*, *MC1R*, *MITF*, *SNAI2*, *SOX10*, *TBX15*, *TYR*). Using a similar partitioning scheme and substitution model as for our initial analyses, we obtain an identical phylogeny (Fig. 3) with similar levels of nodal support (compare Figs. 2 and 3). We conclude that utilising amino acid sequence data consistently resolves the relationship among extant and extinct Rhinocerotidae species in an identical manner, at least for the amino acid sequence data currently available. Our data support the placement of a clade composed of *Dicerorhinus*, *Coelodonta*, and *Stephanorhinus* as a sister group to *Rhinoceros*, to the exclusion of *Ceratotherium* and *Diceros*.

Our phylogenetic tree agrees with the latest Rhinocerotini cladogram based on a morphological character matrix for *Stephanorhinus*, *Coelodonta*, and the extant rhino species in the close relationship between *Stephanorhinus* and *Coelodonta* (Deng et al., 2011). Furthermore, both our and their phylogenetic tree recovers *Ceratotherium* and *Diceros* (two extant African rhinoceros genera) in a clade basal to the other extant rhinoceroses and *Stephanorhinus*/*Coelodonta*. The consensus cladogram presented in that study did not fully resolve the position of *Dicerorhinus* in relation to *Stephanorhinus*/*Coelodonta* and the genus *Rhinoceros*, however. Nevertheless, we see our protein sequence data as providing the first biomolecular support of a close relationship between the genera *Stephanorhinus*, *Coelodonta*, and *Dicerorhinus*. Future analysis of rhinoceros specimens identified to the species level and further development of mass-spectrometry based ancient protein sequencing might make it feasible to test additional hypotheses of the phylogenetic relationships between extinct rhinoceroses at the species level.

ZooMS peptide marker series have previously been shown not to be informative in discriminating between the different Rhinocerotidae species included in this study (Buckley et al., 2009; Welker et al., 2016). This is unfortunate when studying bone assemblages where multiple rhinoceros taxa might be present, such as at Neumark-Nord 2 and Schöningen 13II-4, or in contexts where ZooMS screening results in the presence of several bone specimens now identified as Rhinocerotidae (Welker et al., 2015b, 2017). Although the phylogenetic analysis is not capable of confidently solving the relationship between the genera *Stephanorhinus*, *Coelodonta*, and *Dicerorhinus*, there are several unique amino acid substitutions present within the COL1 sequences obtained for these genera in this study. Therefore, these genera can only be separated based on ancient protein sequence data obtained through LC-MS/MS analysis. Hence, the results of the work presented here could be used as follow-up analysis in cases where site-based ZooMS screening results in the identification of Rhinocerotidae specimens (Welker et al., 2015b, 2017). This is of relevance to understanding the geographic distribution and extinction processes of *Stephanorhinus* and *Coelodonta* (Stuart & Lister, 2012). In contrast, the third member of this clade, *Dicerorhinus*, has survived to the present, albeit critically endangered (Brook et al., 2014; Ripple et al., 2015). Furthermore, it has been proposed that members of both extinct genera occupied slightly different ecological niches (van der Made, 2010; Pushkina, Bocherens & Ziegler, 2014) and hence the precise generic taxonomic attribution through ancient protein analysis of rhinoceros remains at Pleistocene localities is of relevance to a detailed ecological understanding of past faunal communities and hominin activity.

## Conclusion

The Rhinocerotidae represent an enigmatic family of megafauna that comprised an important component of Pleistocene fauna complexes in Eurasia and Africa. Despite past interest, both morphological and DNA based approaches have resulted in different hypotheses on the phylogeny of extant and extinct species. By utilising the recovery of ancient collagenous and NCP sequences for two extinct rhinoceros genera and protein data for five extant species (including all extant genera), we build a protein phylogenetic tree that supports a close phylogenetic relationship between the genera *Stephanorhinus*, *Coelodonta*, and *Dicerorhinus*, as opposed to other extant taxa. We were not able to resolve the order of divergence between these three genera, however. Nevertheless, these genera can be differentiated based on unique amino acid substitutions, enabling taxonomic attributions at the genus-level in future studies. This would be of relevance to understanding the extinction processes of both genera, which are relatively poorly understood, especially for *Stephanorhinus*, as well as more detailed ecological inferences at archaeological and palaeontological sites where ZooMS results in the identification of Rhinocerotidae bone/dental specimens.

## Supplemental Information

10.7717/peerj.3033/supp-1 Supplemental Information 1 Protein sequence information. Origin of protein sequences used for phylogenetic analysis (accession numbers include Genbank, Uniprot and ENSEMBL accession numbers). †PMF40, *SCH3.

10.7717/peerj.3033/supp-2 Supplemental Information 2 Bayesian phylogenetic tree including sample SCH2. The COL1 sequence of SCH2 was very incomplete (30.3%) and no other proteins were detected in this sample. SCH2 should cluster with SCH3, but is placed at the base of sampled Rhinocerotidae. The SCH2 COL1 protein sequences were removed from further consideration once it was clear that no useful phylogenetic information on the position of the genus *Stephanorhinus* could be obtained by including this sample.

10.7717/peerj.3033/supp-3 Supplemental Information 3 Rhinoceros protein sequences generated in this study. Sequences are separated by species/gene. X indicates unknown residues. Leucines (L) have been converted into isoleucines (I) as these two amino acids are isobaric. Telopeptides have been removed for collagen alpha-1(I) and alpha-2(I).

## Additional Information and Declarations

### Data Deposition

The following information was supplied regarding data availability: Database: ProteomeXchange. Accession number: PXD005534.
