# Supplementary figures and images for: Middle Pleistocene protein sequences from the rhinoceros genus Stephanorhinus and the phylogeny of extant and extinct Middle/Late Pleistocene Rhinocerotidae
Source: PeerJ. 2017 Mar 14;5:e3033. doi: 10.7717/peerj.3033 (PMC5354071; doi:10.7717/peerj.3033)

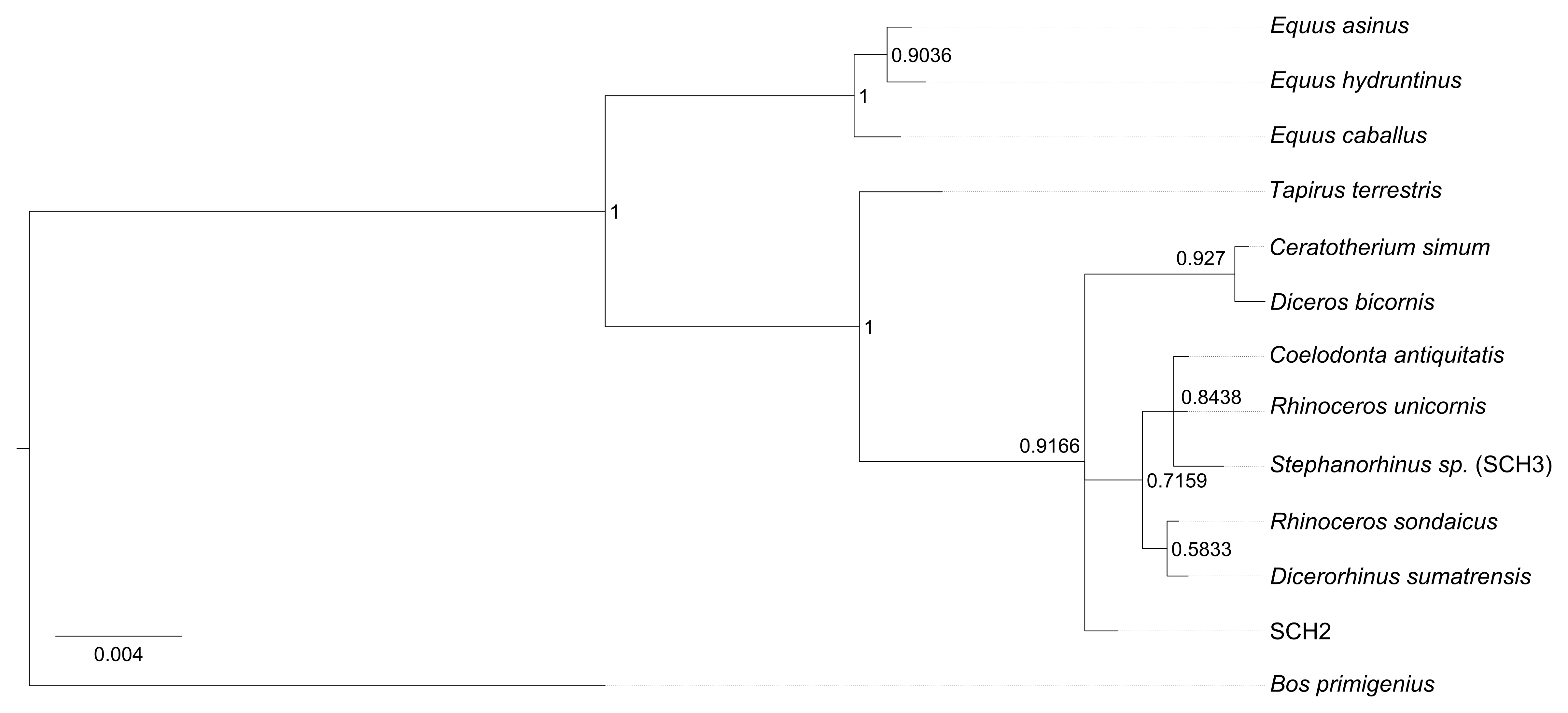

Supplement: Supplemental Information 2 — The COL1 sequence of SCH2 was very incomplete (30.3%) and no other proteins were detected in this sample. SCH2 should cluster with SCH3, but is placed at the base of sampled Rhinocerotidae. The SCH2 COL1 protein sequences were removed from further consideration once it was clear that no useful phylogenetic information on the position of the genus Stephanorhinus could be obtained by including this sample. [file peerj-05-3033-s002.png]
